# Quality of Life Following Early Orthodontic Therapy for Anterior Crossbite: Report of Cases in Twin Boys

**DOI:** 10.1155/2016/3685693

**Published:** 2016-09-22

**Authors:** Eluza Piassi, Leonardo Santos Antunes, Marcia Rejane Thomas Canabarro Andrade, Lívia Azeredo Alves Antunes

**Affiliations:** ^1^Fluminense Federal University, Niterói, RJ, Brazil; ^2^Department of Specific Formation, School of Dentistry, Fluminense Federal University, Nova Friburgo, RJ, Brazil

## Abstract

Anterior crossbite (AC) refers to a condition in which the maxillary anterior teeth are placed lingually in their relationship with the mandibular anterior teeth. This dental condition results in visible incisor differences that are associated with higher levels of dissatisfaction with appearance and have potential to negatively impact on oral health-related quality of life (OHRQoL) of the children. The aim of this paper was to report two cases of interceptive orthodontic treatment of twin children with anterior crossbite and its impact on OHRQoL of these children. Although AC affects negatively psychosocial aspects of OHRQoL of the children, the interceptive orthodontic treatment of children with AC was essential to improve their OHRQoL.

## 1. Introduction

Anterior crossbite (AC) refers to a condition in which the maxillary anterior teeth are placed lingually in their relationship with the mandibular anterior teeth [1]. The prevalence reported in the literature ranges from 0.5% to 11.9% [2–6], depending on the age of the child, ethnic group, and also methods of registration [7].

An anterior crossbite may be regarded as an aesthetic problem and is a malocclusion that is established and is presented in the mixed dentition [8, 9]. Since it is seldom self-corrected, it should be treated as soon as possible, because it can develop into a more severe malocclusion or cause traumatic injury to the periodontal tissues [1]. Moreover, this dental condition results in visible differences in the incisors which are associated with higher levels of dissatisfaction with appearance and may cause a negative impact on oral health-related quality of life (OHRQoL) of the children [10].

Several methods of assessing the OHRQoL of the children are available such as CPQ_11–14_ [11], CPQ_8–10_ [12], Child OIDP [13], ECOHIS [14], and SOHO [15]. The original CPQ_8–10_ was developed and validated in Canada and measures the negative effects of orofacial conditions on the social, emotional, and functional wellbeing of children between 8 and 10 years of age [12]. However, the CPQ_8–10_ has been tested and validated in other countries [16–19].

Several treatment options such as fixed and/or removable appliances, which act directly on the malpositioned teeth, have been devised for early interceptive treatment of the anterior crossbite [20]. A systematic review performed in 2011 disclosed a wide variety of treatment modalities for anterior crossbite correction in the primary dentition or the mixed dentition. Although the level of evidence observed was low to support any technique, there was similarity in the length of time it took to successfully treat anterior crossbites [9].

Therefore, the interceptive orthodontic treatment of anterior crossbite should be carried out in the early mixed dentition with the aim of preventing the development and progression of tooth malalignments and malocclusions and to avoid negative effects on OHRQoL of children [2, 21].

Based on the above mentioned data, the aim of this paper was to report two cases of early orthodontic therapy in twin children with anterior crossbite and its impact on OHRQoL of these children.

## 2. Case Report

Seven-year-old twin boys with anterior crossbite (AC) in permanent teeth were referred to the Preventive/Interceptive Orthodontic Clinic of a Public Dental Service, Rio de Janeiro, Brazil.

A comprehensive clinical examination did not reveal skeletal discrepancies. On manipulation of the mandibles, the incisors obtained an end-to-end relationship, indicative of a dental problem. The canines were in class I occlusion in both cases. About the patient's medical histories, the children were born by cesarean delivery with 36 months of age and normal birth weights (≥2500 g). Both children had breastfeeding for 3 months only and bottle feeding until 3 years of age. They had a dummy-sucking habit until four years of age.

The treatment plan to two children consisted of the interceptive orthodontic treatment with removable plates with springs. In two cases there was sufficient mesiodistal distance to move the upper tooth towards the labial. The legal guardians signed an informed consent form for treatment.

The impact of a children's oral condition on their OHRQoL was measured using the Brazilian version of the Child Perceptions Questionnaire (CPQ_8–10_) [16]. The CPQ_8–10_ is a questionnaire that was developed to assess the impact of oral condition on OHRQoL of children aged 8 to 10 years [16]. This instrument was applied in two different points in time (Table 1): T1: first appointment; T2: after finalized treatment.

The questionnaire contains 25 items organized into 4 health domains: oral symptoms (five items); functional limitations (five items); emotional wellbeing (five items); and social wellbeing (10 items). The items have five Likert response options: “never: 0,” “once or twice: 1,” “sometimes: 2,” “often: 3,” and “every day or almost every day: 4.” The total CPQ_8–10_ score and those for subscales are calculated by summing all the item scores; higher scores indicate that the oral conditions have a greater negative impact on the child's OHRQoL [16]. The question “Why?” was added to the items of each subscale of CPQ_8–10_ when the oral condition caused some impact.

### 2.1. Case  1

**Figure 1:**
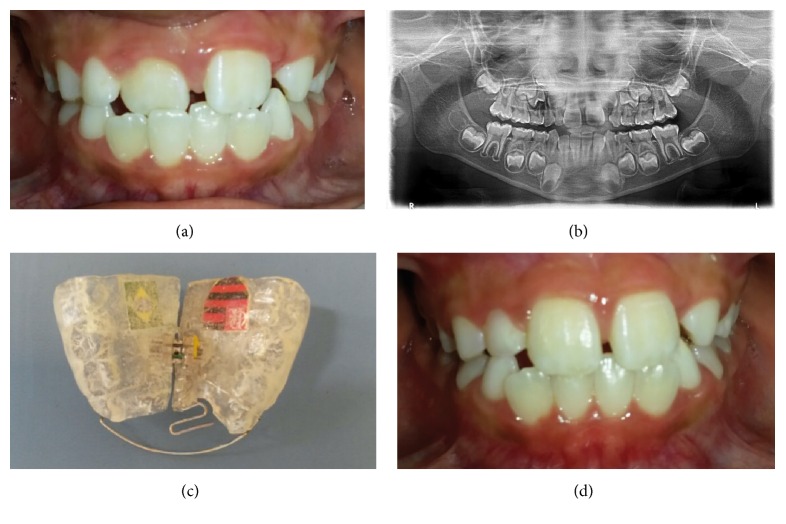
Case  1. (a) Anterior dental crossbite in the upper right central incisor. (b) Panoramic radiograph showing no abnormality. (c) Removable orthodontic appliance with a protrusion spring for incisor in anterior crossbite. (d) Anterior crossbite correction after 3 months.

The first boy presented anterior dental crossbite (ACB) in the upper right central incisor. The clinical exam revealed that the patient was in good health and had no caries experience. A panoramic radiograph revealed no abnormality. Impressions of both arches were taken using alginate and study models made with plaster. The removable appliance was manufactured using acrylic resin, with a protrusion spring for each incisor in anterior crossbite, bilateral occlusal coverage of the posterior teeth, and an expansion screw. The protrusion springs were activated once a month until normal incisor overjet was achieved. The screw was activated during the treatment period only if it was judged to comply with the natural transverse growth of the jaw. The dentist instructed the patient firmly to wear day and night the appliance, except for meals, toothbrushing, and physical activities. The progress was evaluated every 4 weeks. Dental crossbite correction was achieved within the first 3 months of treatment (Figures 1(a), 1(b), 1(c), and 1(d)). The same appliance then served as a passive retainer for a retention period of 3 months.

### 2.2. Case  2

**Figure 2:**
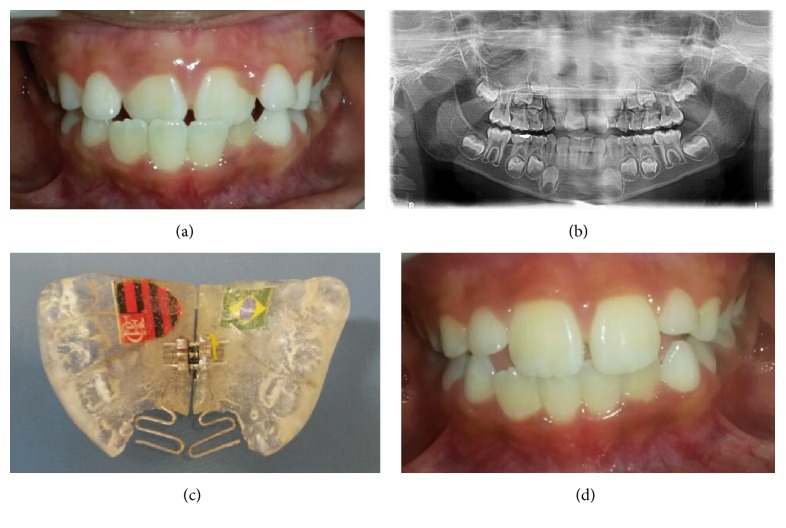
Case  2. (a) Anterior dental crossbite in the two upper central incisors. (b) Panoramic radiograph showing no abnormality. (c) Removable orthodontic appliance with a protrusion spring for each incisor in anterior crossbite. (d) Anterior crossbite correction after 5 months.

Another boy presented ADC in the two upper central incisors. An exam revealed that the patient was in good health and had caries experience. A panoramic radiograph revealed no abnormality. After impression, the same standards of procedure were followed as in the first case. The protrusion springs were activated once a month, until the normal incisor overjet was achieved. The screw was activated during the treatment period only if it was judged to comply with the natural transverse growth of the jaw. The dentist instructed the patient firmly to wear day and night the appliance, except for meals, toothbrushing, and physical activities. The progress was evaluated every 4 weeks. Dental crossbite correction was achieved within the first 5 months of treatment (Figures 2(a), 2(b), 2(c), and 2(d)). The same appliance then served as a passive retainer for a retention period of 3 months.

**Figure 3:**
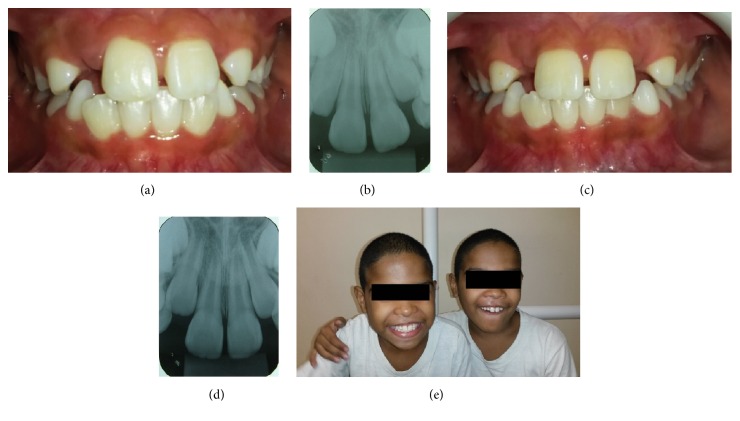
Six months later, after treatment. (Case  1: (a), (b); Case  2: (c), (d)) Central incisors were in normal position and there was no relapse. Periapical radiographs of upper central incisor teeth. There was no pathological condition ((e): twin boys smiling).

Six months later patients were recalled again to assess relapse. In addition, periapical radiographs were taken and no periapical pathology was evident (Figures 3(a), 3(b), 3(c), 3(d), and 3(e)).

## 3. Discussion

Anterior crossbite had negative psychosocial effects on OHRQoL of the twin boys, corroborating with a systematic review that reported that malocclusions have negative effects on OHRQoL, predominantly in the of domains emotional and social wellbeing of children and adolescents [21]. According to Marques et al. [22] and in a recent systematic review [23], malocclusions treatment reduces the impact on children and adolescents' OHRQoL. In this case report it was confirmed; the interceptive orthodontic treatment to correct the anterior crossbite showed the importance of dental esthetics for improvement of a children's OHRQoL.

The differences between CPQ_8–10_ scores before and after treatment confirmed the success of our chosen therapy. Although the twin boys had a negative impact in the same domain of the questionnaire (emotional wellbeing), the scores were different. Only one boy had a negative impact on social wellbeing domain. A possible explanation of these findings could be that the individuals differ in the evaluation of their dental condition and in the perceived psychological consequences [24]. On the other hand, when the children were solicited to answer the question “Why?” to items of subscale that caused impact, they answered: “because the wrong dental position.” Thus, the present case report highlights that malocclusion, especially in the anterior tooth, can compromise a child's psychosocial wellbeing.

In the mixed dentition, anterior crossbite affecting one or more incisors can be successfully corrected by either fixed or removable appliances [20]. Our cases had undergone removable appliance therapy at the mixed dentition stage because Brazilian public dental service does not offer fixed orthodontic treatment.

There is universal agreement that anterior crossbite should be corrected as soon as possible, corresponding to the maturational level of the child [25, 26]. The patient's compliance is a factor to be considered at the removable appliance selection, since this factor is essential for successful treatment [27]. The two cases had satisfactory results, showing that the children and their parents were motivated by therapy.

Another factor to be considered at the appliance selection is the stability of anterior crossbite correction. Previous study concluded that, in the mixed dentition, anterior crossbite affecting one or more incisors can be successfully corrected by either fixed or removable appliances with similar long-term stability [20]. The two patients were recalled again to assess relapse six month later. Clinical examination and radiographs revealed no relapse and no periapical pathology. Periodic recalls of the patients until complete eruption of the permanent teeth are also important because mixed dentition stage is characterized by physiological occlusal changes and, at the same time, new occlusal disorders may occur.

Proper diagnosis and treatment planning can produce the most satisfying results during the mixed dentition stage [28]. Our results highlight the importance of the public dental service that favors the access of the children in mixed dentition stage to orthodontic treatment. Thus, patients with AC should be treated in the early mixed dentition stage to help their satisfaction with appearance and social interaction. Further prospective investigations and studies with a representative sample are needed to confirm the impact of anterior crossbite treatment on OHRQoL of the children.

## 4. Conclusion

Although AC affects negatively psychosocial aspects of OHRQoL of the children, the interceptive orthodontic treatment of children with AC was essential to improve their OHRQoL.

## Figures and Tables

**Table 1:** Total and subscale CPQ_8–10_ scores at two different points in time.

Domains	Case one		Case two	
	T1	T2	T1	T2
*Oral symptoms*				
Toothache	0	0	0	0
Mouth sores	0	0	0	0
Pain upon ingesting cold food	0	0	0	0
Food trapped in teeth	0	0	0	0
Bad smell in mouth	0	0	0	0
*Functional limitations*				
Time for eating	0	0	0	0
Difficulty biting and/or chewing	0	0	0	0
Difficulty eating	0	0	0	0
Trouble talking	0	0	0	0
Difficulty sleeping	0	0	0	0
*Emotional wellbeing*				
Bothered	2	0	1	0
Sad	2	0	2	0
Ashamed	2	0	1	0
Worried	2	0	0	0
Nice	4	0	0	0
*Social wellbeing*				
Child missed school	0	0	0	0
Trouble doing homework	0	0	0	0
Trouble paying attention in class	0	0	0	0
Trouble talking or reading in class	0	0	0	0
Child avoided smiling or laughing	0	0	1	0
Child avoided talking	0	0	0	0
Child avoided other children	0	0	0	0
Staying out of games	0	0	0	0
Victim of name-calling	0	0	0	0
Questions about teeth	0	0	0	0
*Total B-*CPQ_8–10_ * score*	12	0	5	0

T1: first appointment; T2: after finalized treatment.
